# Accumulation of 8-hydroxydeoxyguanosine, L-arginine and Glucose Metabolites by Liver Tumor Cells Are the Important Characteristic Features of Metabolic Syndrome and Non-Alcoholic Steatohepatitis-Associated Hepatocarcinogenesis

**DOI:** 10.3390/ijms21207746

**Published:** 2020-10-20

**Authors:** Anna Kakehashi, Shugo Suzuki, Naomi Ishii, Takahiro Okuno, Yuko Kuwae, Masaki Fujioka, Min Gi, Vasily Stefanov, Hideki Wanibuchi

**Affiliations:** 1 Department of Molecular Pathology, Osaka City University Graduate School of Medicine, Abeno-ku, 1-4-3 Asahi-machi, Osaka 545-8585, Japan; suzuki.shugo@med.osaka-cu.ac.jp (S.S.); m1159070@med.osaka-cu.ac.jp (N.I.); m2026860@med.osaka-cu.ac.jp (T.O.); m2066048@med.osaka-cu.ac.jp (M.F.); mwei@med.osaka-cu.ac.jp (M.G.); wani@med.osaka-cu.ac.jp (H.W.); 2 Department of Diagnostic Pathology, Osaka City University Graduate School of Medicine, Abeno-ku, 1-4-3 Asahi-machi, Osaka 545-8585, Japan; kuwaeyu@med.osaka-cu.ac.jp; 3 Department of Biochemistry, Saint Petersburg State University, 199034 Saint Petersburg, Russia; v.stefanov@spbu.ru

**Keywords:** metabolic syndrome, type 2-diabetes, NASH, HCC, L-arginine, AGR1

## Abstract

To uncover mechanisms and explore novel biomarkers of obesity, type 2 diabetes (T2DM) and nonalcoholic steatohepatitis (NASH)-associated hepatocarcinogenesis, cellular and molecular alterations in the liver, and hepatocellular carcinomas (HCCs) were investigated in NASH model 60-week-old Tsumura, Suzuki, Obese Diabetic (TSOD) mice and NASH HCC patients. Markedly elevated lipid deposition, inflammation, fibrosis, and peroxisome proliferation in the liver, preneoplastic lesions, and HCCs of TSOD mice were accompanied by accumulation of polysaccharides in the cellular cytoplasm and nuclei and increase of oxidative DNA damage marker, 8-hydroxydeoxyguanosine (8-OHdG) formation in the liver and altered foci. Metabolomics of TSOD mice HCCs demonstrated significant elevation of the concentration of amino acid L-arginine, phosphocreatine, *S*-adenosylmethionine/*S*-adenosylhomocysteine ratio, adenylate, and guanylate energy charges in coordination with tremendous rise of glucose metabolites, mostly fructose 1,6-diphosphate. L-arginine accumulation in HCCs was associated with significant under-expression of arginase 1 (ARG1), suppression of the urea cycle, methionine and putrescine degradation pathways, activation of Ser and Thr kinase Akt AKT, phosphoinositide 3-kinase (PI3K), extracellular signal-regulated kinase 1/2 (ERK1/2) kinases, β-catenin, mammalian target of rapamycin (mTOR), and cell proliferation. Furthermore, clinicopathological analysis in 20 metabolic syndrome/NASH and 80 HCV-positive HCC patients demonstrated significant correlation of negative ARG1 expression with poor tumor differentiation, higher pathological stage, and significant decrease of survival in metabolic syndrome/NASH-associated HCC patients, thus indicating that ARG1 could become a potential marker for NASH HCC. From these results, formation of oxidative stress and 8-OHdG in the DNA and elevation of glucose metabolites and L-arginine due to ARG1 suppression in mice liver cells are the important characteristics of T2DM/NASH-associated hepatocarcinogenesis, which may take part in activating oxidative stress resistance, synthesis of phosphocreatine, cell signaling, methylation, and proliferation.

## 1. Introduction

Nonalcoholic fatty liver disease (NAFLD) and steatohepatitis (NASH) are now very common chronic hepatic conditions predisposing to hepatocellular carcinoma (HCC) development. According to the recent “multiple-parallel-hits hypothesis”, NASH could be observed in both obese and non-obese patients and is caused by multiple events, such as abnormal metabolism and accumulation of lipids, mitochondrial dysfunction, and oxidative and endoplasmic reticulum stresses [1]. Recent studies have demonstrated that tissues other than the liver could also be involved in NASH development due to endotoxin-induced inflammation in the gut and bile duct and adipocytokines, leptin and adiponectin derived from adipose tissue [2,3]. Our recent proteome analysis of NASH biopsies and HCCs of patients with metabolic syndrome and diabetes mellitus type 2 (T2DM) demonstrated coordinated activation of SMAD3-transforming growth factor-β (TGF-β), β-catenin, nuclear factor (erythroid-derived 2)-like 2 (Nrf2), sterol regulatory element-binding protein and liver X receptor α and nuclear receptor-interacting protein 1 [4]. These changes, along with inhibition of tumor protein p53 and peroxisome proliferating receptors, suggested their involvement in development of steatosis and fibrosis, generation of oxidative stress, rise of cell proliferation, and suppression of apoptosis in NASH hepatocarcinogenesis [4]. However, the concrete mechanisms of progression from NASH to HCC are still not well understood. Due to difficulties with clinical human liver samples, NASH animal models are of importance as they may help to understand functional and molecular mechanisms associated with specific histopathological features, such as hepatocyte ballooning, steatosis, inflammation, and fibrosis.

Recent data demonstrated involvement of various genetic and environmental factors in NASH development. Animal models of NASH-associated HCC include feeding of rodents with methionine and/or choline-deficient high-fat diets [5], all of which have clear differences and limitations. One well-developed NASH animal model considered closely reflecting the human situation (is the Tsumura, Suzuki, Obese Diabetic (TSOD) mouse metabolic syndrome model, established by selective breeding of ddY mice [6]. As compared with age-matched control Tsumura, Suzuki, Non-Obese (TSNO) mice, TSOD mice are characterized by hyperphagia, hyperinsulinemia, hypertriglyceridemia, hypercholesterolemia, and hyperleptinemia, and they develop T2DM before 4 months of age, associated with increase of body weight, impaired glucose tolerance, insulin resistance, impaired insulin secretion, and hyperglycemia [6]. Insulin resistance observed in TSOD mice is likely due to disturbance of insulin-initiated translocation of glucose transporter type 4, non-insulin-dependent diabetes (Nidd) 4, Nidd5, and Nidd6 [7]. TSOD mice feature NASH histopathological characteristics, such as microvesicular fat and hepatocellular ballooning, inflammatory cell infiltration, formation of Mallory bodies from 4 months of age, and development of liver tumors at 12 months [8]. It has been further demonstrated that impaired glucose tolerance, hyperglycemia, and inflammation observed in TSOD mice is preceded by oxidative stress. However, concrete mechanisms of hepatocarcinogenesis in TSOD mice still need to be detailed.

In the present study, a combination of histopathological, electron microscopic, metabolome, proteome, and immunohistochemical analyses was applied to investigate the characteristic features of NASH-associated hepatocarcinogenesis in TSOD mice. Furthermore, clinicopathological analysis was performed with human metabolic syndrome/NASH-associated and HCV-positive (HCV^+^) HCCs.

## 2. Results

### 2.1. General Conditions, Clinical Signs, Body and Organ Weights

Significant elevation of body weights, food and water intakes, relative liver, and significant decrease of relative kidneys weights were found in TSOD mice (Supplementary Table S1). TSOD mice were obese, developed insulin resistance and exhibited early stages of the NASH phenotype. Thus, starting from 8 weeks of age, fasting blood glucose, as well as urine glucose levels, were found significantly elevated. Moreover, at 60 weeks of age, significant rise of blood levels of triglycerides, total cholesterol, free fatty acids, aspartate aminotransferase (AST), alanine transaminase (ALT), alkaline phosphatase (ALP), and albumin was observed. The results indicated development of fatty change and abnormalities of the liver in TSOD mice but not in TSNO age-matched littermates.

### 2.2. Histopathological and Electron Microscopic Observations of Liver

**Figure 1:**
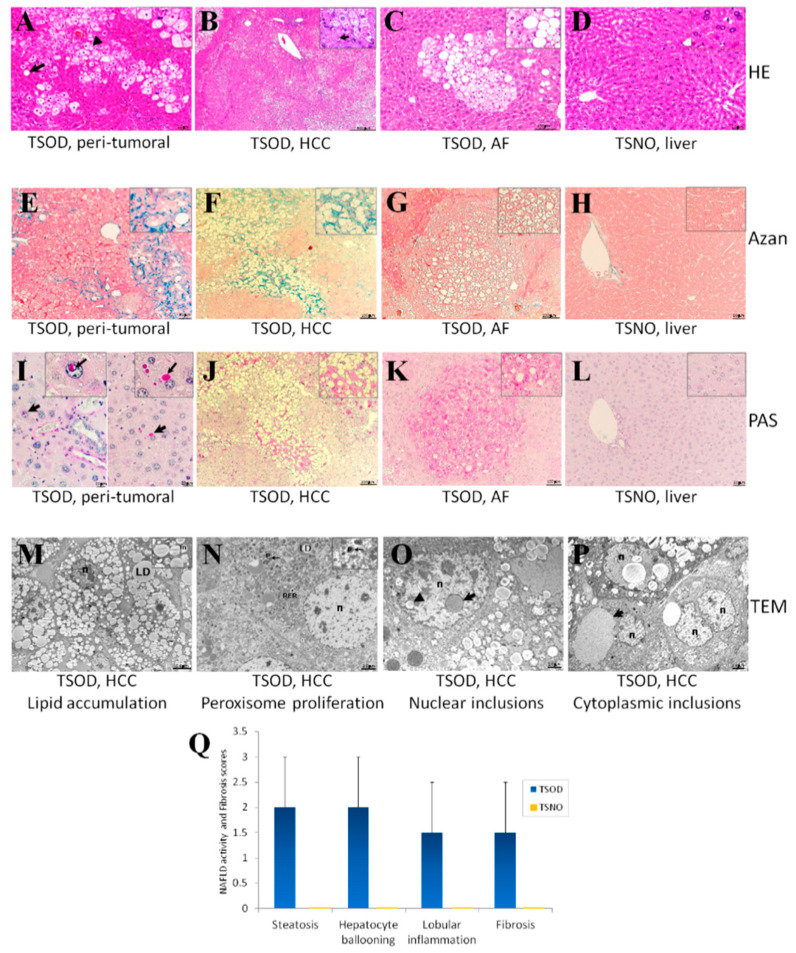
Representative histopathological (H&E) (**A)**–(**D**), Azan (**E**)–(**H**), Periodic acid-Schiff (PAS) (**I**)–(**L**) staining, TEM (**M**)**–**(**P**) features, and NAFLD activity (NAS) and fibrosis scores (**Q**) in 60-week-old Tsumura, Suzuki, Obese Diabetic (TSOD) mice livers (**A**,**C**,**E**,**G**,**I**,**K**) and hepatocellular carcinomas (HCCs) (**B**,**F**,**J**,**M**–**P**), and control Tsumura, Suzuki, Non-Obese (TSNO_ mouse liver (**D**,**H**,**L**). Lipid degeneration, fatty change (arrows), mild inflammation (**A**), vacuolated (lipids-accumulated) HCCs with eosinophilic droplets (arrow) (**B**) and altered foci (AF) (**C**) in TSOD mouse livers. Azan-positive regions (fibrosis) TSOD liver (**E**), HCC, AF and PAS-positive nuclear and cytoplasmic inclusions (arrows) (**I**), HCC (**J**), and AF (**K**) were observed in TSOD mice livers. TEM analysis (**M)**–(**P**) demonstrated strongly increased number of lipid droplets (LD), peroxisomes (p), numerous nuclear (n), cytoplasmic inclusions with grey homogenous material (arrows) and lipids (triangle arrows), and mitochondria (m) in HCCs of TSOD mice. Bars: 200 µm (**B**); 100 µm (**C**,**F**,**G**,**J**,**K**); 50 µm (**A**,**D**,**E**,**H**,**L**); 20 µm (**I**); 5.6 µm (**M**); 2.5 µm (**N**,**P**); 2.0 µm (**O**).

We have previously reported significant increase of liver tumors (HCC and hepatocellular adenoma (HCA)) incidences (TSOD, 81%, n = 16; TSNO, 0%, n = 5, *p* < 0.05) and multiplicities (TSOD: 1.7 ± 1.4 no./mouse; TSNO: 0.0 ± 0.0 no./mouse) in TSOD mice at 60 weeks of age [9]. Representative pictures showing histopathological changes in TSOD mouse livers are shown in Figure 1. NASH-associated changes, such as fatty degeneration, inflammatory cell infiltration, and hepatocellular ballooning (Figure 1A), as well as formation of vacuolated/lipid-accumulated HCCs (Figure 1C) and altered foci (AF) (Figure 1D), were found. In addition, Azan staining demonstrated development of fibrosis in the livers (Figure 1E), HCCs (Figure 1F), and AFs (Figure 1G) TSOD but not the TSNO mice livers (Figure 1H). Similarly, PAS-positive glycogenated nuclear and cytoplasmic inclusions (Figure 1I), HCCs (Figure 1J), as well as PAS-positive AF (Figure 1K), were observed in the livers of TSOD but not the TSNO mice.

TEM microphotographs of the HCC cells found in TSOD mice are shown in Figure 1M–P. Marked accumulation of large and small lipid-rich cellular organelles (lipid droplets (LD)) and hepatocyte ballooning were observed in HCCs and HCAs of TSOD mice (Figure 1M). In tumor cells with large numbers of LD, the number of mitochondria (m) was not high (Figure 1M). Furthermore, peroxisomes (p) and rough endoplasmic reticulum (RER) were prominent (Figure 1N). Interestingly, liver tumor cells of TSOD mice often contained nuclear (Figure 1O) and cytoplasmic (Figure 1P) inclusions with lipids and/or grey homogenous material.

### 2.3. Metabolome Alterations

The results of metabolome analysis in the liver tumors and livers of TSOD and TSNO mice are summarized in Table 1. Interestingly, analysis of the metabolome changes in TSOD mouse HCCs demonstrated drastic 13.5-fold elevation of amino acid L-arginine in comparison with TSNO mice liver. In NASH liver tissue of TSOD mice, L-arginine levels were also increased significantly, apparently associated with elevation of alanine and argininosuccinate. No significant differences in citrulline/ornithine ratios were observed in HCCs and livers of TSOD mice as compared to TSNO controls. However, the citrulline/L-arginine ratio was significantly decreased in HCCs (25-fold) and livers (2-fold) of TSOD mice, indicating significant shift in metabolism from citrulline to L-arginine. Furthermore, a strong trend for increase of the L-arginine/ornithine ratio (24.6-fold) in TSOD HCCs was evident. In TSOD mice HCCs, a significant increase was observed for phosphocreatine (8-fold), which is synthesized from L-arginine, and is a rapidly mobilizable reserve of high-energy phosphates to recycle adenosine triphosphate (ATP) and an important energy pool.

Marked increase of glucose metabolites including glucose 1-phosphate (16-fold), glucose 6-phosphate (17-fold), galactose 1-phosphate (25-fold), fructose 6-phosphate (14-fold) and extremely high levels of 1,6-fructose diphosphate (390-fold) were found in TSOD HCCs as compared to the TSNO control livers. A trend for increase of glucose metabolites was also observed in TSOD mouse NASH livers. Rise of L-arginine and glucose metabolites levels in TSOD mouse HCCs was associated with significant elevation of lactic acid in HCCs and surrounding liver tissues (Table 1). Furthermore, significant elevation of ATP and guanosine-5'-triphosphate (GTP) and decrease of adenosine monophosphate (AMP) and guanosine monophasphate (GMP) was found in TSOD mice HCCs. These changes indicated significant increases of adenylate and guanylate energy charges in HCCs of TSOD mice. Furthermore, a trend for decrease of glycerol 3-phosphate (glycerol 3-P)/dihydroxyacetone phosphate (DHAP) ratio in TSOD HCCs was apparent, indirectly reflecting the ratio of reduced and oxidized forms of nicotinamide adenine dinucleotide (NADH/NAD^+^) and energy supply.

Interestingly, we observed significant increase of *S*-adenosylmethionine (SAM) in HCCs of TSOD mice, associated with decreases of *S*-adenosylhomocysteine (SAH), methionine, homoserine and choline levels. A significant 4.7-fold elevation of the SAM/SAH ratio was detected, which is a marker of tissue methylation activity [10]. Furthermore, significant decrease of glycine (2.4-fold) and N-dimethylglycine levels was observed in TSOD mice HCCs. Next, significant increases of polyamines, putrescine, and spermidine, but not spermine, as well as putrescine/spermidine and spermidine/spermine ratios, in the livers and strong trends for increase in HCCs of TSOD mice were found, indicating a shift from spermine and spermidine to putrescine in the livers and liver tumors of TSOD NASH model mice. Furthermore, a trend for increase of AcCoA and significant elevation of CoA, acetoacetyl CoA, and succinic acid, which are the important molecules involved in lipid biogenesis or the tricarboxylic acid (TCA) cycle, were observed in TSOD mice livers. On the other hand, levels of aspartic acid in the NASH livers were significantly low. In TSOD HCCs, those changes were much less pronounced and the only significantly elevated metabolite was succinic acid, pointing to suppression of the TCA cycle. Lastly, cystathionine was found significantly elevated in HCCs of TSOD mice. Moreover, a significant decrease of oxidized form of glutathione (GSSG) was associated with a trend for increase of its reduced form (GSH) and the GSH/GSSG ratio in TSOD NASH HCCs, possibly indicative of activation of mechanisms of resistance of tumor cells to oxidative stress.

### 2.4. HMT, Functional, Canonical Pathway and Up-Stream Regulator Analyses

**Figure 2:**
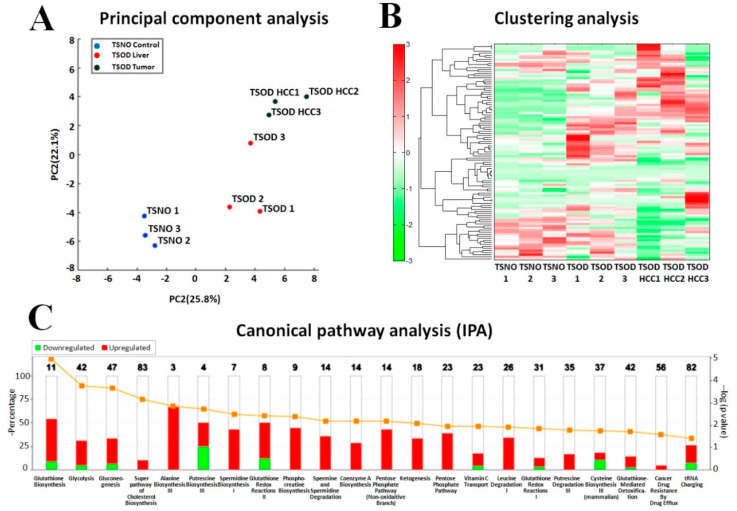
Alterations detected by the metabolome and IPA analyses in the livers and tumors of TSOD mice. Principal component analysis (**A**), clustering (**B**), and canonical pathways (**C**) analyses.

Results of principal component analysis (PCA), hierarchical clustering, canonical and up-stream regulator analyses by Human Metabolome Technologies (HMT) and Ingenuity Pathway Analysis (IPA) are presented in Figure 2. PCA successfully separated out different clusters corresponding to TSNO control, TSOD livers and TSOD HCC samples (Figure 2A). In clustering analysis, the patterns of metabolite expression in TSOD livers and HCCs were notably different from that of TSNO control mice livers (Figure 2B).

Examination of altered functional and canonical pathways by IPA on the basis of metabolome analysis demonstrated that many metabolites with altered expression in TSOD mice HCCs were involved in the processes of liver cholestasis, synthesis of reactive oxygen species (ROS), glutathione biosynthesis and redox reactions and regulation of hepatocytes proliferation, cell death and regeneration (Figure 2C). Furthermore, alterations were detected in glycolysis I, gluconeogenesis I, pentose phosphate pathway, transport of amino acids, phosphocreatine, cholesterol, CoA, putrescine, alanine and cysteine biosynthesis, spermine, spermidine, putrescine and leucine degradation, ketogenesis, activation of lymphocytes, and tRNA charging (Figure 2C).

Results of IPA upstream regulator analysis based on metabolome analysis predicted significant activation of D-glucose (z-score = 2.186) and creatine metabolism (z-score = 2.000) dependent of elevation of numerous glucose and creatine metabolites in TSOD mice HCCs (Supplementary Table S2).

### 2.5. Comparative Proteome and Metabolome Alterations in TSOD Mice

A list of differentially expressed proteins in HCCs and livers of TSOD mice detected by LC-Ms/Ms analysis is presented in the Supplementary Table S3. Comparative analysis of altered metabolites and proteins by IPA indicated that elevation of numerous glucose metabolites was associated with increase of expression of enzymes involved in glycolysis, gluconeogenesis and lactate synthesis from pyruvate, such as glucose-6-phosphate isomerase, fructose-1,6-bisphosphatase, and lactate dehydrogenase A, in NASH-associated TSOD mice HCCs.

Elevation of L-arginine was associated with significant inhibition of urea cycle enzymes, including ARG1, argininosuccinate lyase (ASL), argininosuccinate synthase 1 (ASS1) and ornithine carbamoyltransferase (OTC). Expression of ARG1, responsible for the formation of ornithine from arginine, was strongly suppressed in TSOD mice HCCs and also inhibited in NASH livers. Controversially, in the liver tissue of TSOD mice, expression of ASS1 was upregulated, signifying activation of L-arginine synthesis, while other urea cycle enzymes were not altered.

Significant increase of SAM/SAH ratio was coordinated with inhibition of enzymes involved in the degradation of methionine, including methionine adenosyltransferase 1A (MAT1A), glycine N-methyltransferase (GNMT), betaine-homocysteine S-methyltransferase 1 (BHMT), and adenosylhomocysteinase (AHCY). In addition, elevation of GSH appeared linked with decrease of glutathione peroxidase (GPX), glutathione S-transferase kappa 1 and microsomal glutathione S-transferase 1.

In addition to alteration of enzymes mentioned above, numerous proteins involved in lipid metabolism, actin cytoskeleton and intermediate filament organization, protein folding and cell signaling were elevated in HCCs of TSOD mice (Supplementary Table S3). Thus, proteins participating in lipopolysaccharide/interleukin 1 (LPS/IL-1) -mediated inhibition of retinoid X receptor, RXR) function, liver X receptor (LXR)/RXR activation, phospholipase C signaling, oxidation-reduction process, Nrf2 oxidative stress response, peroxisomal proliferation, pregnane X receptor (PXR)/RXR signaling and protein folding were significantly overexpressed. In contrast, proteins involved in fatty acid β-oxidation in mitochondria, such as acyl-CoA oxidase 1 and 2, were under-expressed. Up-stream regulator analysis from proteome data demonstrated activation (z-score ≥ 2.0) of insulin, like growth factor 1 (IGF-1), tumor necrosis factor (TNF), interleukin 10 receptor subunit alpha (IL10RA), Nrf2, rapamycin-insensitive companion of mTOR (RICTOR), versican (VCAN), adenosine A2a receptor (ADORA2A), β-catenin (CTNNB1), single-minded homolog 1 (SIM1), and hepatocyte nuclear factor 1A (HNF1A), but no significant activity of oncogenes. Proteome analysis of HCCs in 45% choline-deficient, L-amino acid-defined high-fat diet (CDAHFD)-fed mice demonstrated activation of Nrf2, TNF and HNF4A, but no activation of IGF-1, RICTOR, β-catenin, and other upstream regulators which activation was detected in TSOD mice HCCs.

### 2.6. Alterations to Cell Proliferation (Ki67) and Apoptosis (TUNEL) and 8-hydroxydeoxyguanosine (8-OHdG) Formation

**Figure 3:**
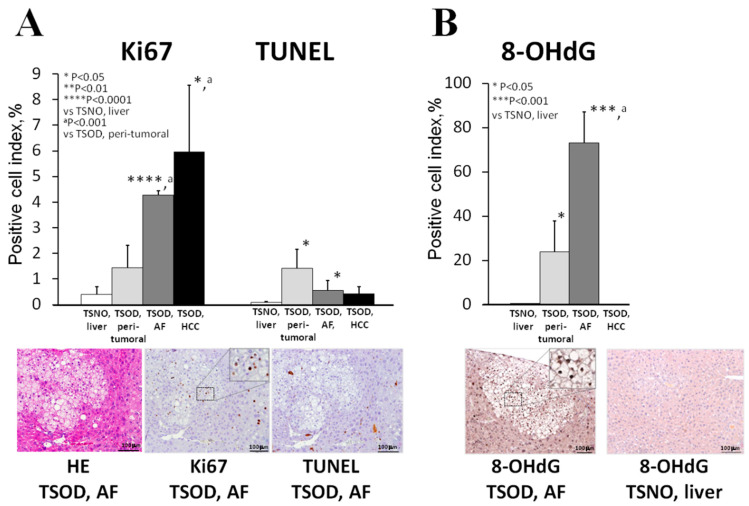
Alteration of cell proliferation (Ki67) and apoptosis (TUNEL) (**A**) and 8-hydroxydeoxyguanosine (8-OHdG) formation (**B**) in livers, altered foci (AF) and tumors of TSOD and TSNO mice.

Significant elevation and trends for increase of Ki67-positive cell ratio for hepatocytes were observed in TSOD mice AF, HCCs and livers, respectively, as compared to TSNO mouse livers (Figure 3A). Furthermore, in AF and HCCs, cell proliferation indices were significantly increased as compared to surrounding liver tissue of TSOD mice. The number of apoptotic cells was found significantly elevated in TSOD NASH livers and AF in comparison to TSNO mice. In TSOD mice HCCs and AF, apoptotic positive cell number tended to decrease, as compared to the surrounding liver tissue (Figure 3A). In addition, immunohistochemical analysis of oxidative DNA damage marker, 8-OHdG, showed significant increases in TSOD mice livers and AF, respectively, and very low levels of expression in the control TSNO livers and TSOD mice HCCs (Figure 3B).

### 2.7. Immunohistochemical Assessment of Urea Cycle Enzymes, Phosphokinases and β-catenin

**Figure 4:**
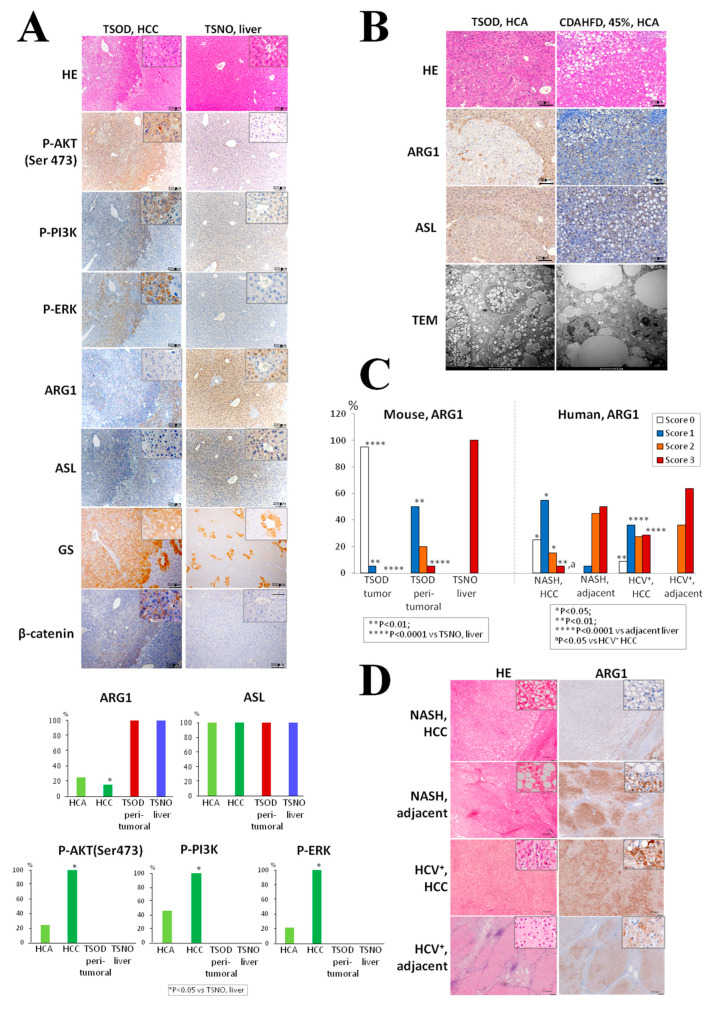
Representative pictures and quantitative immunohistochemical analysis of P-AKT (Ser473), P-PI3K, phospho-ERK1/2 (P-ERK), arginase 1 (ARG1), argininosuccinate lyase (ASL), glutamine synthase (GS) and β-catenin in TSOD mice HCCs (**A**). P-AKT(Ser 473), P-PI3K, P-ERK1/2, and GS were strongly overexpressed in the HCCs of TSOD mice. ARG1, ASL expression, and TEM findings in TSOD and 45% choline-deficient, L-amino acid-defined high-fat diet (CDAHFD)-treated mice HCAs (42-weeks) (**B**). Note significant suppression of ARG1 and slight decrease of ASL expression in TSOD, but high expression in 45% CDAHFD-treated mice HCA, lipid degeneration, and ballooning of TSOD mice, and accumulation of large size lipid droplets in 45% CDAHFD-treated mice tumors. ARG1 and ASL were highly expressed in the livers of C57Bl/6J control age-mached littermates, peri-tumoral, and tumor tissues of CDAHFD-treated mice. Immunohistochemical findings for ARG1 in mouse and human livers and HCCs (**C**). Note strong inhibition of AGR1 expression in TSOD mouse tumors and nonalcoholic steatohepatitis (NASH) HCCs. AGR1-negative and positive cases of NASH-associated and HCV^+^ human HCCs, respectively (**D**).

Representative results of immunohistochemical examination of phosphokinases, ARG1 and ASL in TSOD, TSNO and 45% CDAHFD-treated mice (42 weeks) are presented in Figure 4.

Incidences of P-AKT(Ser473) (100%, score 3+), P-PI3K (100%, score 3+), and P-ERK1/2 (100%, score 3+)-positive HCCs were significantly increased in TSOD mice as compared to respective TSNO control animals and TSOD surrounding liver tissues (Figure 4A,C). In TSOD HCAs, only a trend for increase was observed. Thus, P-AKT(Ser473) was elevated in 13% of HCAs (scores 3+ and 1+, respectively), and both P-PI3K and P-ERK1/2 were diffusely overexpressed (score 3+) in 22.7% of HCAs of TSOD mice.

Significant downregulation of ARG1 was observed in all TSOD mice HCCs and HCAs (95%, score 0), with only one HCA positive (5%, score 1+) (Figure 4B,C). Significantly decreased ARG1 expression (score 3+) was also observed in the liver of TSOD mice as compared to TSNO mice (Figure 4A,C). Controversially, only slight suppression of ASL expression in TSOD mice liver tumors and AF was demonstrated (Figure 4A,B). Interestingly, suppression of ARG1 in TSOD mice HCCs was highly correlated with overexpression of glutamine synthetase (GS) in liver pericentral areas and HCCs and also coordinated with elevation of cytoplasmic and nuclear β-catenin. β-catenin has been previously demonstrated to control ARG1 and GS expression in the metabolic liver and tumor zonation [11] (Figure 4A). In 45% CDAHFD-fed C57Bl/6J mice, ARG1 and ASL were elevated in HCCs and HCAs, as well as in the peri-tumoral livers and liver tissues, of non-treated control age-matched littermates of the same background. Thus, the differences in the expression of urea cycle enzymes were obvious in diabetes-related NASH-TSOD and CDAHFD diet models.

### 2.8. Alterations to mRNA Expression of DNA Repair Enzymes

**Figure 5:**
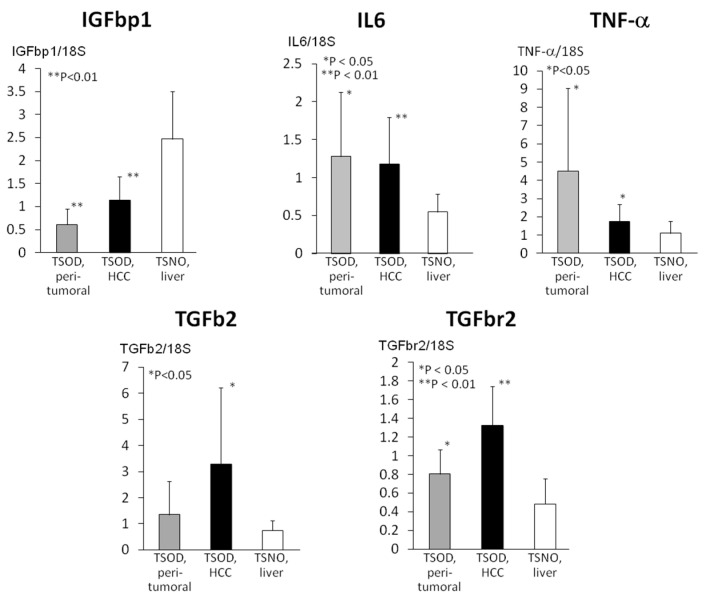
Insulin-like growth factor binding protein 1 (*IGFbp1*), interleukin 6 (*IL6*), tumor necrosis factor alpha *(TNF-α*), transforming growth factor b2 (*TGFb2*), and transforming growth factor binding receptor 2 (*TGFbr2*) mRNA expression changes in TSOD mice livers and tumors. Note the significant decrease of IGFbp1 in TSOD mice livers and HCCs, associated with significant elevation of markers of inflammation (IL6, TNF-α), and fibrosis (TGFb2 and TGFbr2).

Insulin-like growth factor binding protein 1 (IGFbp1) mRNA expression was significantly inhibited in the livers and HCCs of TSOD mice (Figure 5).

In contrast with *IGFbp1* mRNA changes, inflammation markers interleukin 6 (*IL6*) and tumor necrosis factor alpha *(TNF-α)* mRNA levels were significantly elevated in TSOD mice livers and HCCs as compared to the control TSNO mice liver. Furthermore, markers of fibrosis, transforming growth factor b2 (TGFb2) and transforming growth factor binding receptor 2 were elevated in TSOD mice livers, being more pronounced in HCCs.

### 2.9. ARG1 Expression in Human Metabolic Syndrome/NASH-Associated and HCV^+^ HCCs and Association with Clinicopathological Variables

In the specimens of 20 NASH HCC patients examined, ARG1 was negative in 5 (25% (score 0)), weakly-expressed in 11 (55% (score 1+)), moderately expressed in 3 (15% (score 2+)), and strongly-expressed in only 1 (5% (score 3+)) (Figure 4C,D). In adjacent livers, no negative cases were found, while ARG1 was weakly expressed in 1 case (5%), moderately in 9 (45%) and strongly expressed in 10 (50%). In HCV^+^ HCCs, ARG1 was negative in 7 (7.5% (score 0)), weakly-expressed in 30 (37.5% (score 1+)), moderately-expressed in 22 (27.5% (score 2+)) and strongly-expressed in 22 (27.5% (score 3+) cases. In adjacent livers of HCV^+^ HCC patients, no negative and weakly-positive ARG1 cases found, while moderately and strongly-positive cases were 29 (36.3% (score 2+)) and 51 (63.8% (score 3+) patients, respectively. Thus, in metabolic syndrome/NASH-associated and HCV^+^ HCCs, negative/weakly-positive and moderate/high ARG1stainings predominated, respectively (Figure 4C). The significant suppression of highly positive (score 3) ARG1 expression was found in NASH HCCs in comparison with HCV^+^ HCCs (*p* < 0.05). Nevertheless, in both HCC types, significant increases of incidences of negative and weakly-expressed and decreases of strongly expressed ARG1 were observed, as compared to adjacent livers.

**Figure 6:**
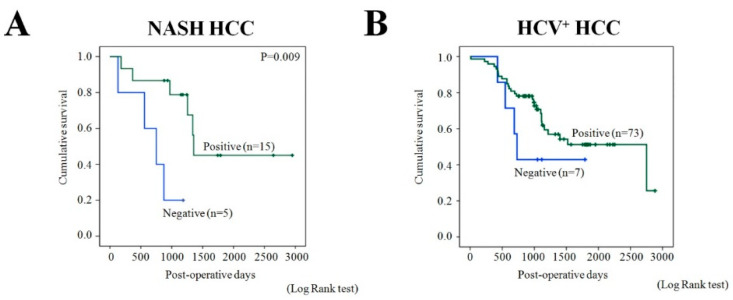
Association of ARG1 expression with NASH-associated (**A**) and HCV^+^ (**B**) patients’ cumulative survival.

Clinicopathological analysis demonstrated significant correlation of negative ARG1 expression with poor tumor differentiation (*p* = 0.04) in human metabolic syndrome/NASH-associated but not in HCV^+^ HCCs (Table 2). In both metabolic syndrome/NASH and HCV-associated patients, significant correlation of ARG1 suppression and higher pathological stage was detected (*p* = 0.02 and *p* = 0.04, respectively). Furthermore, significant association of ARG1 positive expression with increase of serum AST and ALP was found in HCV^+^ HCC patients (Table 2). Interestingly, results of univariate survival analysis demonstrated significant association of ARG1 negativity with decrease of cumulative survival in NASH HCC (log-rank test; *p* = 0.009) as compared with positive expression (Figure 6).

In HCV^+^ HCC patients, no significant difference between ARG1 negativity and decrease of cumulative survival was found.

## 3. Discussion

Results of the present study demonstrated that development of HCCs in the livers of T2DM/NASH model TSOD mice at 60-weeks of age was associated with increases of ballooning (vacuolar) degeneration, steatosis, inflammation, fibrosis, activated actin and intermediate cytoskeleton organization, peroxisome proliferation, formation of cytoplasmic eosinophilic bodies and glycogenated nuclei, marked accumulation of oxidative DNA damage marker 8-OHdG in the liver and AF, and significant elevation of glucose metabolites (especially fructose-1,6-bisphosphate) and L-arginine. Elevation of serum AST, ALT, ALP, albumin, total cholesterol, free fatty acids, and TG also likely to play a role. Those changes are the important characteristics of T2DM/NASH-associated hepatocarcinogenesis in TSOD mice.

NAFLD and NASH are often associated with insulin resistance, glucotoxicity and accompanied by an increase in cholesterol, triglycerides, insulin, as well as decrease in adiponectin [12]. Furthermore, elevated glucose levels and hyperinsulinemia stimulate *de novo* lipogenesis in the liver by activation and up-regulation of lipogenesis transcriptional factors. Previous studies revealed that body weights, plasma total cholesterol level and lipid oxidation products in TSOD mice are increased at 5 weeks of age. Obesity and insulin resistance developed at around 12 weeks, histopathological features of NASH could be detected from 16 weeks, while expression of Cyp2c and Cyp3a, pregnane X receptor (PXR) and peroxisome proliferator-activated receptor-gamma coactivator-1alpha mRNA are elevated at 28-weeks of age [8,13]. It has been previously suggested that increased oxidative stress could be an initial event that triggers the development of diabetes in the TSOD mouse, but the concrete mechanisms were unknown [8]. Here, we found that generation of peroxisomes and oxidative DNA damage marker, 8-OHdG, in the livers of TSOD mice were associated with rise of inflammation (TNF-α, IL6), fibrosis (TGFb2, TGFbr2) markers, and cell proliferation. Therefore, it is conceivable that formation of 8-OHdG in the DNA of TSOD mice liver cells is an early event in T2DM/NASH-associated hepatocarcinogenesis. In addition, fructose 1,6-diphosphate was previously shown to exert cytoprotective activity and to act as an antioxidant due to its ability to bind soluble iron, sequester Fe(II) and prevent its transformation to the insoluble Fe(III), which generates reactive ROS via Fenton reactions [14]. Previously, marked iron accumulation was found in the spleen and liver of TSOD mice during early stages of NASH, significantly correlating with its severity [8]. Thus, in the TSOD mice livers, elevation of fructose 1,6-diphosphate in response to oxidative stress could exert cytoprotective and antioxidant effects via binding with iron, and take part in neoplastic transformation. Furthermore, increase of the GSH/GSSG ratio, which could be due to activation of cysteine biosynthesis, also reflects increase of antioxidant activity in the HCCs. Both processes are likely to be involved in development of oxidative stress resistance in tumor cells.

**Figure 7:**
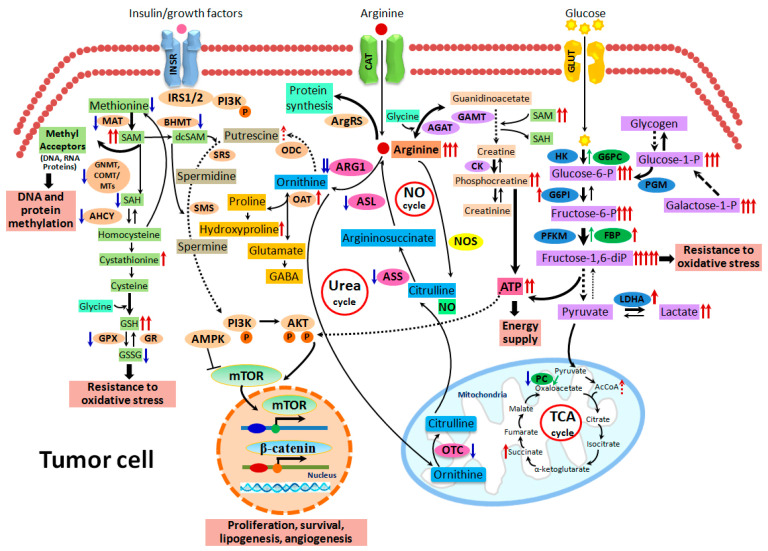
Metabolome and proteome alterations in the liver tumor cells of TSOD mice. Marked changes of glycolytic, gluconeogenic, fructose, galactose, pyruvate, methionine, glutathione and creatine metabolism in HCCs of TSOD mice were closely associated with L-arginine accumulation, and activation of mTOR and β-catenin signaling pathways.

In our study, metabolic pathway analysis further revealed significant perturbation of glycolytic, gluconeogenic, fructose, galactose, pyruvate and creatine metabolism in HCCs of TSOD mice, closely associated with L-arginine accumulation and increased insulin sensitivity of tumor cells (Figure 7).

Marked elevation of fructose 1,6-diphosphate and increase in the glucose 6-P/ribose-5-P ratio in HCCs reflected higher level of glycolysis over pentose phosphate cycle. Previous studies demonstrated that whole-body insulin sensitivity could be improved by supplementation with medium doses (9 g/day) of L-arginine to patients with visceral obesity and/or T2DM, as well as high fat diet-treated rodents, leading to increase of glucose uptake, normalized hepatic arterial and portal blood flows, as well as microcirculation, increased hepatic tissue oxyhemoglobin, and further enhanced cellular resistance to oxidative stress [15]. Interestingly, SAM was also highly elevated in HCCs of TSOD mice. More than 120 methyltransferases are known to be dependent on SAM which is transformed into SAH, so that the intracellular SAM/SAH ratio reflects the levels of methylation in the tissue [16]. Thus, the observed SAM/SAH elevation signified activation of methylation in TSOD mice HCCs. It has been reported that the disturbance in the regulation of SAM synthesis and catabolism resulting in the shift from SAH to SAM in the mouse liver can lead to fatty liver disease and to the development of HCC [17]. A lack of methionine has been shown to induce a posttranscriptional down-regulation of cystathionine β-synthase and may contribute to the maintenance of SAM and SAH levels [18]. It is well-known that L-arginine could be methylated by type I and II protein L-arginine methyltransferases, affecting protein interactions and participation in numerous cellular processes [19].

L-arginine is an important amino acid, progenitor of many proteins and numerous important biomolecules, whose physiological requirement in healthy people and animals is covered by its endogenous synthesis and intake with the food. In the normal healthy organism, food-derived L-arginine is absorbed across the small intestine and is transported to the liver, where it is converted to L-ornithine and urea via binuclear manganese metalloenzyme ARG1 (urea cycle) and partially used as a substrate for nitric oxide (NO) synthesis carried out by NO synthases (NOS) (Figure 7) [20]. L-ornithine can also be metabolized via ornithine aminotransferase (OAT) to L-proline, which is used for collagen production and deposition [21]. Thus, L-arginine synthesis may occur from proline, glutamine or argininosuccinate in the urea cycle. In mammals, L-arginine is used for protein synthesis via arginyl t-RNA synthetase, production of creatine via L-arginine:glycine amidinotransferase and guanidinoacetate N-methyltransferase (Figure 7), and possibly L-agmatine via mitochondrial arginine decarboxylase (ADC) [22]. L-arginine levels in blood plasma have been found to be increased with age and catabolic disease states, such as sepsis and injury [23,24]. From our results, L-arginine is a very important feature of T2DM/NASH hepatocarcinogenesis, closely associated with activation of glucose uptake, creatine metabolism, and formation of resistance to oxidative stress by liver tumor cells.

In the present study, we evaluated and compared ARG1 expression in the livers and tumors developed in different NASH mouse models. From previous studies, in high fat diet rodent NASH model, decreased NO bioavailability coupled with changes in two key NO metabolic enzymes, suppression of activated eNOS and increase of ARG1, has been reported in steatotic livers [25]. However, in the present T2DM/NASH model, no elevation of ARG1 was observed, pointing to differences in mechanisms of hepatocarcinogenesis in high fat diet and T2DM/NASH models. In addition, accordingly to the data obtained from IPA and Gene Expression Omnibus (GEO) database (GSE63067), except for CDAHFD diet model, enzymes of urea cycle including ARG1, ASL, ASS1, and OTC are similarly downregulated in TSOD, Stelic Animal Model (STAM), MAT1, and GNMT knockout (KO) mice NASH models, which are all characterized by altered SAM production [26]. Thus, elevation of SAM and suppression of ARG1 could be interrelated. In the present study, ARG1 downregulation in TSOD mice HCCs was further coordinated with activation of β-catenin, AKT, PI3K, and ERK1/2, elevation of GS and cellular proliferation. Furthermore, we have previously demonstrated activation of mTOR, IGF1, TNF, and Nrf2 in TSOD HCCs [9]. β-catenin has been reported as an important regulator of metabolic liver zonation, affecting the expression of GS and urea cycle enzymes including ARG1, thus, the correlative controversial changes of ARG1 and GS are likely to be a result of activation of Wnt/β-catenin/T-cell factor (TCF) signaling pathway [27]. In respect to mTOR activation, large doses (35 g/day) of L-arginine were previously found to stimulate the release of growth hormone, which could activate mTORC1 and induce protein synthesis in HCC [28]. Thus, the accumulation of L-arginine in the liver of TSOD mice may affect the mTOR signaling, as well.

Suppression of ARG1 detected in NASH-associated HCCs, livers of TSOD mice and human NASH-associated HCCs is an interesting novel finding of the present study. ARG1 expression could be demonstrated to be high in HCV^+^ HCC, but low in metabolic syndrome/NASH-associated tumors. Furthermore, poorly differentiated NASH tumors contained higher percentages of ARG1 negative cells, suggesting that ARG negativity is mainly correlated with poor HCC differentiation, which could be the reason of poor survival. Controversially, in HCV^+^ HCCs, no association with poor differentiation and survival of patients was found, pointing to differences in L-arginine metabolism in these liver cancers. Association of low AGR1 expression with higher pathological stage in NASH and HCV^+^ HCC patients indicated, that AGR1 expression is gradually decreasing during HCCs development. Previous research demonstrated that metabolism of L-arginine to ornithine and increased ARG1 activity in the liver contributed to HCV-mediated stimulation of hepatocellular growth due to activation of NO-mediated cell death [29]. From the literature, the existence of an “arginine paradox” has been suggested regarding roles of L-arginine and ARG1 in tumor development [30,31]. From our results, this could be due to the differences in the origin of HCC and the mechanisms underlying hepatocarcinogenesis in NASH and virus-associated tumors.

In conclusion, enhanced lipid and cholesterol biogenesis, inflammation, and fibrosis in the liver of TSOD mice was associated with formation of oxidative stress and 8-OHdG generation in the DNA of liver preneoplastic cells. Furthermore, marked accumulation of L-arginine, glucose metabolites, phosphocreatine, and SAM are proven to be the very important metabolic characteristics of T2DM/NASH-associated liver tumors, which are likely to be related to activation of oxidative stress resistance, cellular methylation, β-catenin, mTOR pathways, and cell proliferation. From our results, downregulation of ARG1 may become an important marker associated with decrease of survival in metabolic syndrome and NASH-associated human liver cancer patients.

## 4. Materials and Methods

### 4.1. Chemicals

Chemicals were obtained from Sigma (St. Louis, MO, USA) or Wako Pure Chemicals Industries (Osaka, Japan).

### 4.2. Experimental Design, Liver Tissue, and Tumor Samples

Liver, liver tumor samples for present histopathological, metabolome, proteome, immunohistochemical and transmissional electron microscopic (TEM) analyses were obtained from our previous study [9], which was performed in accordance with the Guidelines of the National Institute of Health and Public Health Service Policy on the Humane Use and Care of Laboratory Animals, and approved by the Ethics Committee of the Institutional Animal Care and Use Committee of Osaka City University Graduate School of Medicine, Osaka, Japan (No. 14AE). In short, 20 male 5-week-old TSOD and 5 TSNO (control) mice also of the ddy strain but without the metabolic syndrome were obtained from Japan SLC, Inc. (Shizuoka, Hamamatsu, Japan), housed in plastic cages (5 mice/cage) with wood chips for bedding and quarantined for 1 week before the start of the study. Animals were housed in an animal facility at a constant temperature of 23 ± 1 °C and relative humidity of 44 ± 5% and given free access to tap water and food (Oriental MF pellet diet, Oriental Yeast Co., Tokyo, Japan) for 54 weeks. Mouse body weights and fasting blood and urine glucose levels were measured before autopsy performed at 60 weeks of age. After overnight fasting, blood was collected via the abdominal aorta and biochemical analysis of serum samples was performed using an automatic analyzer (Olympus AJ-5200, Tokyo, Japan). Livers and tumors were immediately subjected to macroscopic analysis and fixed in 10% buffered formalin, Bouin or 2% glutaraldehyde, 2% paraformaldehyde, and 2% osmium tetroxide solutions, and prepared for histological, immunohistochemical, TEM analyses, periodic acid-Schiff stain (PAS) and Azan stain to detect the polysaccharides and collagen fibers, respectively. For the comparison of ARG1 expression in different NASH mouse models, we obtained formalin-fixed and paraffin-embedded samples from C57Bl/6J mice treated with choline-deficient, L-amino acid-defined, 45 kcal% high-fat diet (45% CDAHFD) containing 0.1% methionine (Charles River, Kanagawa, Japan) for 42 weeks [9]. Firstly, AGR1 and ASL immunohistochemical results were compared to the control age-matched mice of the same background (C57BL/6J) and only then with TSOD mice.

### 4.3. Metabolome and Proteome Analyses

Metabolome measurements were carried out through a facility service at Human Metabolome Technologies, Inc. (HMT) (Tsuruoka, Japan) [9]. Frozen liver tumors (HCCs) and surrounding livers (approximately 50 mg each) of 3 TSOD mice and 3 TSNO mice were plunged into 1500 µL of 50% acetonitrile/Milli-Q water containing internal standards (H3304-1002, HMT, Tsuruoka, Japan) at 0 °C in order to inactivate enzymes. The tissues were homogenized thrice at 1500 rpm for 120 s using a tissue homogenizer (Micro Smash MS100R, Tomy Digital Biology Co., Ltd., Tokyo, Japan) and then the homogenate was centrifuged at 2300× *g* and 4 °C for 5 min. Eight hundred microliters of upper aqueous layer was centrifugally filtered through a Millipore 5-kDa cutoff filter at 9100× *g* and 4 °C for 120 min to remove proteins. The filtrate was centrifugally concentrated and re-suspended in 50 µL of Milli-Q water for CE-MS analysis. Peaks were acquired in both positive and negative ion mode (separate injections) and metabolites were identified according to chromatographic retention time (migration time (MT)), molecular weight (*m*/*z*), and MS/MS fragmentation [32,33,34]. Peaks detected by CE-TOFMS (Agilent Technologies, Santa Clara, CA, USA) and CE-MS/MS were extracted using automatic integration software (MasterHands, Keio University, Tsuruoka, Japan) and MassHunter Quantitative Analysis B.04.00 (Agilent Technologies, Santa Clara, CA, USA), respectively, in order to obtain peak information including *m/z*, migration time (MT), and peak area. The peaks were annotated with putative metabolites from the HMT metabolite database based on their MTs in CE and *m/z* values determined by TOFMS and MS/MS. In addition, concentrations of metabolites were calculated by normalizing the peak area of each metabolite with respect to the area of the internal standard and by using standard curves, which were obtained by three-point calibrations. The data were applied to the principal component analysis (PCA) (PeakStat software, HMT, Tsuruoka, Japan). Hierarchical cluster analysis (HCA) was performed by HMT proprietary software, SampleStat. For each analysis, data peak standardization was performed (µ = 0, σ = 1). Metabolites were identified by the HMT data library (HMT, Tsuruoka, Japan). Metabolic pathway analyses were performed with VANTED (Visualization and Analysis of Networks containing Experimental Data). Data were also subjected to Kyoto Encyclopedia of Genes and Genomes (KEGG) database (http://www.genome.jp/kegg/) and Ingenulty Pathway Analysis (IPA) (Ingenuity Systems, Mountain View, CA, USA) for the analysis of altered upstream regulators and signaling pathways. With IPA analysis, z-scores above or lower 2 were considered significant.

### 4.4. Human Tissue Samples and Patients

The study with human specimens was approved by the Osaka City University Graduate School of Medicine Ethics Committee (Osaka, Japan) and performed according to the principles of Declaration of Helsinki with informed consent was obtained from all patients (No. 2263). The present analyses covered tissue specimens obtained from 20 NASH and 80 HCV+ patients with primary HCCs undergoing operations at Osaka City University Hospital (Osaka, Japan) from January 2006 to December 2016. All cases were histologically proven and were enrolled to assess the clinical significance of expression levels of arginase 1 (ARG1) in resected liver specimens of primary HCCs. Information on the clinicopathological characteristics of patients obtained from medical records are presented in Supplementary Table S4. Histological analyses and pathological diagnoses were performed by at least two pathologists from the pathology department in our hospital according to the Japanese classification of HCC [35] and the criteria for general guidelines for primary liver cancer of the American Joint Committee on Cancer/International Union against Cancer staging systems [36] and the Liver Cancer Study Group of Japan [35]. The validated system proposed by the NASH Clinical Research Network was applied for scoring NASH histopathological features [37].

### 4.5. Immunohistochemical Examination

A standard ABC method using a Vectastain Elite ABC Kit (PK-6102; Vector Laboratories, Burlingame, CA, USA) was employed to perform the immunohistochemistry on formalin-fixed paraffin-embedded liver sections. Target proteins were visualized by development of a peroxidase reaction using a solution of 3,3′-diaminobenzidine tetrahydrochloride (Dojndo Laboratories, Kumamoto, Japan). The List of antibodies applied overnight at 4 °C is presented in Supplementary Table S4. Immunohistochemical detection of apoptosis was performed using TUNEL assay (ApopTag Peroxidase In Situ Apoptosis Detection Kit S7100, Sigma-Aldrich, Co. St. Louis, MA, USA) by the supplied protocol. Immunohistochemical procedures were optimized by testing different antigen retrieval methods and negative controls.

### 4.6. Real-Time Quantitative PCR

Total RNA from mouse livers and tumors was isolated using the Isogen (Nippon Gene, Toyama, Japan). Oligo-dT primer was used for the reverse transcription of 2 µg of total RNA. TaqMan Gene Expression Assays (4351372) (Applied Biosystems, Tokyo, Japan), TaqMan probes, and primes sets were applied for the real-time quantitative PCR of *IGFbp1* (Mm 00515154_m1)*, IL6* (Mm 00446190_m1), *TNF-α* (Mm 00443258_m1), *TGFb2* (Mm 00436955_m1), *TGFbr2* (Mm 03024091_m1) mRNA. For the internal control, eukaryotic 18S rRNA(4319413E) (Applied biosystems, Tokyo, Japan) was applied. Relative numbers of target mRNA and *18S* RNA transcripts were shown as a result. PCR analyses were done in triplicates.

### 4.7. Statistical Analysis

The significance of differences between mean values was analyzed by the two-tailed Student *t*-test using the StatLight-2000(C) program (Yukms Corp., Kanagawa, Japan). Results were plotted as mean ± standard deviation (SD) values. For the statistical analysis of metabolite data, Ingenuity Pathways Analysis (IPA; QIAGEN Bioinformatics, Redwood City, CA, USA) (upstream regulator and pathway analyses) was used. To reveal clustering effects hierarchical clustering analysis was performed. SPSS statistics version 19.0 (SPSS Inc., Chicago, IL, USA) was applied to analyze associations between ARG1 immunohistochemical expression in human HCCs and clinicopathological variables were evaluated using Pearson’s Chi-square test. The Kaplan-Meier method with SPSS software was used for the calculation of survival curves from the day of surgery to relapse, death or the last follow-up observation, and difference in cumulative survival curves was assessed with the Log rank test. In all applied analyses, *p*-values < 0.05 were considered statistically significant.

## Figures and Tables

**Table 1:** Altered metabolites and metabolic parameters in the livers and tumors of TSOD mice.

Metabolic Parameter	Category	KEGG ID	Name	TSOD *p*-Tum. vs. TSNO Liv.			TSOD HCC vs. TSNO Liv.		
				Ratio	*p*-Value		Ratio	*p*-Value	
Glucose Metabolites	Glycolysis	C00103	Glucose 1-phosphate (G1P)	9.13	0.07		16.09	0.05	
	Gluconeogenesis	C00092	Glucose 6-phosphate (G6P)	9.71	0.08		17.44	0.03	*
		C00085	Fructose 6-phosphate (F6P)	7.81	0.07		14.09	0.04	*
		C00354	Fructose 1,6-diphosphate (F1,6P)	21.57	0.13		389.92	0.02	*
		C00446	Galactose 1-phosphate (Gal1P)	23.56	0.07		24.95	0.00	***
Arginine Biosynthesis		C00062	Arginine (Arg)	1.72	0.03	*	13.48	0.04	*
and Catabolism	Arginase	C00077	Ornithine	0.78	0.12		0.89	0.74	
Urea cycle	NOS	C00327	Citrulline	0.75	0.35		0.42	0.11	
	Argininosuccinase	C03406	ArgSuccinate	1.60	0.02	*	1.79	0.39	
		C02305	Phosphocreatine	3.53	0.27		7.95	0.02	*
			Citrulline/Ornithine	0.93	0.88		0.72	0.54	
			Citrulline/L-Arginine	0.44	0.04	*	0.04	0.02	*
			L-Arginine/Ornithine	2.21	0.00	**	24.26	0.13	
Adenylate Energy Charge	Energy State	C00020	AMP	0.83	0.30		0.62	0.04	*
		C00008	ADP	1.46	0.28		1.46	0.24	
		C00002	ATP	2.44	0.29		4.36	0.00	***
		-	Adenylate Energy Charge	1.76	0.16		2.45	0.04	*
Total Adenylate	Purine B/C	-	Total Adenylate	0.93	0.66		0.76	0.14	
Guanylate Energy Charge	Energy State	C00144	GMP	0.83	0.20		0.81	0.04	*
		C00035	GDP	1.11	0.28		1.14	0.21	
		C00044	GTP	1.22	0.32		1.50	0.04	*
		-	Guanylate Energy Charge	1.38	0.13		1.56	0.01	**
Total Guanylate			Total Guanylate	0.86	0.23		0.85	0.06	
G6P/R5P	Glycolysis, PPP	C00092	Glucose 6-phosphate (G6P)	9.71	0.08		17.44	0.03	*
		C00117	Ribose 5-phosphate (R5P)	1.05	0.91		0.45	0.19	
		-	G6P/R5P	10.41	0.20		39.25	0.12	
Glycerol 3-P/DHAP	ORP	C00093	Glycerol 3-phosphate (Glycerol 3-P)	1.63	0.01	**	1.43	0.17	
		C00111	Dihydroxyacetone phosphate (DHAP)	6.13	0.13		33.73	0.14	
		-	Glycerol 3-phosphate/DHAP	0.32	0.32		0.07	0.23	
Lactate/Pyruvate	Anaerobic Glycolysis	C00186	Lactic acid	3.84	0.01	**	4.27	0.04	*
	Gluconeogenesis	C00022	Pyruvic acid	3.19	0.09		2.95	0.18	
			Lactate/Pyruvate	1.31	0.50		1.79	0.40	
NADH/NAD+	ORP	C00003	NAD^+^	1.76	0.17		1.12	0.80	
		C00004	NADH	1.03	0.47		0.98	0.74	
		-	NADH/NAD+	0.42	0.35		0.69	0.60	
NADPH/NADP+	OS, FA Synthesis	C00006	NADP^+^	1.70	0.25		1.19	0.71	
		C00005	NADPH	1.01	0.82		1.08	0.58	
			NADPH/NADP+	0.45	0.35		0.74	0.65	
GSH/GSSG	Oxidative Stress	C00127	Glutathione (GSSG)	0.81	0.11		0.55	0.03	*
		C00051	Glutathione (GSH)	89.14	0.06		154.63	0.17	
		-	GSH/GSSG	82.36	0.11		229.05	0.20	
	Cysteine, Glutathione Biosynthesis	C00542	Cystathionine	1.94	0.08		1.87	0.00	**
		-	Total Glutathione	1.18	0.02	*	1.20	0.52	
Putrescine/Spermidine	Cancer, Aging	C00134	Putrescine	3.59	0.02	*	11.19	0.09	
Spermidine/Spermine		C00315	Spermidine	1.55	0.01	*	1.96	0.10	
		C00750	Spermine	1.28	0.37		0.56	0.40	
		-	Putrescine/Spermidine	2.33	0.00	**	5.71	0.06	
		-	Spermidine/Spermine	1.23	0.24		3.81	0.06	
	Putrescine, Glutamate MET	C00334	γ-Aminobutyric acid (GABA)	1.83	0.003	**	1.10	0.58	
SAM/SAH	Methylation Status	C00021	*S*-Adenosylhomocysteine (SAH)	0.89	0.43		0.55	0.01	*
		C00019	*S*-Adenosylmethionine (SAM)	1.82	0.13		2.62	0.01	*
		-	SAM/SAH	2.13	0.24		4.66	0.01	**
	Choline, Glycine MET, Methylation	C01026	N,N-Dimethylglycine (DMG)	1.01	0.72		0.89	0.04	*
	Lipid MET, Methylation	C00114	Choline	0.70	0.04	*	0.30	0.02	*
	Glycine MET, Methylation	C00719	Betaine	2.09	0.19		2.90	0.29	
TCA cycle		C00024	Acetyl CoA (AcCoA)	14.23	0.09		11.77	0.20	
		C00010	CoA	5.10	0.02	*	2.67	0.13	
		C00332	Acetoacetyl CoA	5.25	0.02	*	1.95	0.17	
		C00158	Citric acid	0.81	0.55		1.10	0.67	
		C00042	Succinic acid	2.93	0.01	*	2.76	0.02	*
		C00122	Fumaric acid	1.04	0.76		0.96	0.87	
Malate/Asp		C00149	Malic acid (Malate)	0.66	0.30		1.20	0.75	
		C00049	Aspartic acid (Asp)	0.59	0.01	**	0.72	0.30	
		-	Malate/Asp	1.16	0.76		1.45	0.42	
Amino acids (A/A)	A/A B/C, I/E	C_0002	Glycine (Gly)	0.53	0.01	*	0.42	0.01	**
		C_0004	Alanine (Ala)	2.07	0.00	**	1.61	0.15	
		C00147	Adenine	1.29	0.06		1.52	0.17	
		C_0014	Valine (Val)	1.40	0.09		1.06	0.66	
		C_0026	Aspartic acid (Asp)	0.59	0.01	**	0.72	0.30	
		C_0034	Methionine (Met)	0.72	0.16		0.40	0.00	**
		C00263	Homoserine	1.22	0.40		0.57	0.04	*
		C_0036	Histidine (His)	1.22	0.10		0.90	0.45	
		C_0038	Phenylalanine (Phe)	1.43	0.11		1.02	0.82	
		C_0039	Arginine (Arg)	1.72	0.03	*	13.48	0.04	*
		C00148	Proline (Pro)	1.26	0.34		1.25	0.21	
		C01015	Hydroxyproline	1.25	0.11		1.43	0.04	*
		C_0044	Tryptophane (Trp)	1.40	0.09		1.38	0.09	
		-	Total Essential A/A	1.20	0.20		0.89	0.36	
		-	Total Non-essential A/A	1.23	0.07		1.03	0.61	
		-	Total Glucogenic A/A	1.23	0.07		1.01	0.79	
		-	Total Ketogenic A/A	1.17	0.28		0.87	0.42	
		-	Total Aromatic A/A	1.26	0.24		0.93	0.40	
		-	Total Glutamic acid-related A/A	1.48	0.23		1.26	0.15	
		-	Total Acetyl-CoA-related A/A	1.23	0.19		0.92	0.57	
		-	Total Fumarate-related A/A	1.25	0.26		0.88	0.15	
		-	Total Branched Chain A/A (BCAA)	1.36	0.12		1.05	0.65	
		-	Total Pyr-related A/A	1.13	0.13		0.87	0.18	
		-	Total Succinyl CoA-related A/A	1.31	0.14		0.98	0.84	
		-	Total Oxaloacetate-related A/A	0.66	0.01	**	0.77	0.25	
		-	Total A/A	1.23	0.08		1.00	0.95	
Fisher’s Ratio	Liver Function, A/A MET	-	Fisher’s Ratio	1.08	0.14		1.12	0.05	a)

Data are Mean value (Student *t*-test (Welch corr.); * *p* < 0.05; ** *p* < 0.01; *** *p* < 0.001; a) *p* = 0.05). A/A, amino acids; B/C, Biosynthesis/Catabolism; DHAP, Dihydroxyacetone phosphate; ADP, adenosine diphosphate; GDP, guanosine diphosphate; MET, metabolism; ORP, Oxidation-Reduction Potential; PPP, Pentose Phosphate Pathway. I/E, Influx/Efflux.

**Table 2:** Correlation between ARG1 expression and clinicopathological variables.

Factors	Metabolic Syndrome/NASH HCC				HCV^+^ HCC			
	(No. Patients/%)				(No. Patients/%)			
ARG1	Total	Positive (*n* = 15)	Negative (*n* = 5)	*p*	Total	Positive (*n* = 73)	Negative (*n* = 7)	*p*
Age								
>70	9	7(47%)	2(40%)	0.80	53	56(77%)	6(86%)	0.59
≤70	11	8(53%)	3(60%)		27	17(23%)	1(14%)	
Gender								
Male	15	11(73%)	4(80%)	0.77	55	51(70%)	4(57%)	0.49
Female	5	4(27%)	1(20%)		25	22(30%)	3(43%)	
Smoking								
Smoker	8	7(47%)	1(20%)	0.29	45	33(45%)	4(57%)	0.91
Non-smoker	12	8(53%)	4(80%)		35	40(55%)	3(43%)	
Drinking								
Drinker	0	0(0%)	0(0%)	-	29	43(59%)	2(29%)	0.52
Non-drinker	20	15(100%)	5(100%)		51	30(41%)	5(71%)	
BMI								
≤24	3	3(20%)	0(0%)	0.28	66	61(84%)	5(71%)	0.42
>25	17	12(80%)	5(100%)		14	12(16%)	2(29%)	
Diabetes								
Positive	14	10(67%)	4(80%)	0.57	19	54(74%)	7(100%)	0.12
Negative	6	5(33%)	1(20%)		61	19(26%)	0(0%)	
Tumor size								
≤2 cm^3^	5	5(33%)	0(0%)	0.14	49	45(62%)	4(57%)	0.82
>2 cm^3^	15	10(67%)	5(100%)		31	28(38%)	3(43%)	
T category								
T1	2	2(13%)	0(0%)	0.39	27	20(27%)	2(29%)	0.95
T2-T4	18	13(87%)	5(100%)		53	53(73%)	5(71%)	
pB								
Positive	1	1(7%)	0(0%)	0.51	1	1(1%)	0(0%)	0.76
Negative	19	14(93%)	5(100%)		79	72(99%)	7(100%)	
Venous invasion								
Positive	6	4(27%)	1(20%)	0.37	21	18(25%)	2(29%)	0.82
Negative	14	11(73%)	4(80%)		59	55(75%)	5(71%)	
Infiltration to capsule								
Positive	13	9(60%)	4(80%)	0.42	47	44(60%)	3(43%)	0.37
Negative	7	6(40%)	1(20%)		33	29(40%)	4(57%)	
Differentiation ^†^								
Well&Moderate	12	11(73%)	1(20%)	0.04	43	39(53%)	4(57%)	0.85
Poorly	8	4(27%)	4(80%)		37	34(47%)	3(43%)	
Pathological Stage ^‡^								
I&II&III	17	14(93%)	3(60%)	0.02	78	72(99%)	6(86%)	0.04
IV	3	1(7%)	2(40%)		2	1(1%)	1(14%)	
Cirrhosis								
Stage 1&2	14	12(80%)	2(40%)	0.09	38	33(45%)	5(71%)	0.18
Stage 3&4	6	3(20%)	3(60%)		42	40(55%)	2(29%)	
AST								
13–33 IU/L;	12	10(67%)	2(40%)	0.29	20	15(21%)	5(71%)	0.003
>34 IU/L	8	5(33%)	3(60%)		60	58(79%)	2(29%)	
ALT								
6-27 IU/L	10	7(47%)	3(60%)	0.61	29	25(34%)	4(57%)	0.23
>28 IU/L	10	8(53%)	2(40%)		51	48(66%)	3(43%)	
ALP								
115-359 IU/L	8	9(62%)	4(33%)	0.38	54	47(64%)	7(100%)	0.05
>360 IU/L	12	5(38%)	2(67%)		26	26(36%)	0(0%)	

Pearson’s Chi square test; pT factor: pathological T factor; pM factor: pathological M factor; ^†^ Poorly vs. Well & Moderately differentiated; ^‡^ Stage IV vs. Stages I&II&III. No lymph node metastasis was detected in all patients.

## Supplementary Materials

The following are available online at https://www.mdpi.com/1422-0067/21/20/7746/s1, Table S1: Organ weights and blood biochemistry analysis in TSOD and TSNO mice; Table S2: Up-stream regulator analysis in TSOD mice tumors by IPA; Table S3: Differentially-expressed proteins in the liver tumors and livers of TSOD mice; Table S4: List of Antibodies.
