# Supplementary material for: Accumulation of 8-hydroxydeoxyguanosine, L-arginine and Glucose Metabolites by Liver Tumor Cells Are the Important Characteristic Features of Metabolic Syndrome and Non-Alcoholic Steatohepatitis-Associated Hepatocarcinogenesis
Source: Int J Mol Sci. 2020 Oct 20;21(20):7746. doi: 10.3390/ijms21207746 (PMC7594076; doi:10.3390/ijms21207746)
Supplement: Supplementary file 1 [file ijms-21-07746-s001.zip › Table S4.docx]

| Table S5. List of Antibodies. | | |
| --- | --- | --- |
| Antibody | Dilution | Number/Company |
| Rabbit monoclonal antibody against arginase 1 (ARG1) | 1:100 | ab124917, Abcam Co., Japan |
| Rabbit polyclonal antibody argininosuccinate lyase (ASL) | 1:50 | ab97370, Abcam Co., Japan |
| Rabbit polyclonal antibodies against β-catenin (β-cat) | 1:100 | ab32572, Abcam Co., Japan |
| Rabbit monoclonal antibody against glutamine synthetase (GS) | 1:500 | Ab176562, Abcam Co., Japan |
| Rabbit monoclonal antibody against P-Akt (Ser473) | 1:100 | # 4060 (D9E), Cell Signaling Technologies Inc. |
| Rabbit polyclonal antibodies against P-PI3K | 1:100 | ab86714, Abcam Co., Japan |
| Rabbit polyclonal antibodies against P- ERK1/2 (Thr202) | 1:300 | #9101 (D13.14.4E, Cell signaling Technology Inc. |
| Rabbit monoclonal antibody against Ki67 | 1:300 | ab16667, Abcam Co., Japan |
| Mouse monoclonal antibody against 8-OHdG | 1:500 | N45.1 , Japan Institute for the control of Aging, Fukuroi, Japan |
