# Supplementary material for: Accumulation of 8-hydroxydeoxyguanosine, L-arginine and Glucose Metabolites by Liver Tumor Cells Are the Important Characteristic Features of Metabolic Syndrome and Non-Alcoholic Steatohepatitis-Associated Hepatocarcinogenesis
Source: Int J Mol Sci. 2020 Oct 20;21(20):7746. doi: 10.3390/ijms21207746 (PMC7594076; doi:10.3390/ijms21207746)
Supplement: Supplementary file 1 [file ijms-21-07746-s001.zip › Table S3.docx]

| Table S3. Differentially-expressed proteins in the liver tumors and livers of TSOD mice. | | | | | | | | |
| --- | --- | --- | --- | --- | --- | --- | --- | --- |
| Protein | Accession # | TSOD tumor vs TSNO liver | |  | TSOD liver vs TSNO liver | | Location | Function |
|  |  | Ratio | P value |  | Ratio | P value |  |  |
| actin gamma 1 (ACTG1) | 6752954 | 2.023 | 0.0000 |  | 2.146 | 0.0000 | ACS, FA | ACO, CJA |
| Rho GDP dissociation inhibitor alpha (ARHGDIA) | 31982030 | 1.776 | 0.0034 |  | 1.380 | 0.0161 | CM, C, EPR, ES | ACO, AP |
| myosin regulatory light chain 12B (MYL12A) | 21728376 | 1.697 | 0.0001 |  | 1.240 | 0.0412 | ACS | ACO |
| myosin heavy chain 9 (MYH9) | 114326446 | 1.437 | 0.0000 |  | 1.208 | 0.0003 | ACS | ACO |
| apolipoprotein A-I (APOA1) | 160333304 | 8.791 | 0.0000 |  | 1.152 | 0.0332 | ES, PM, EPR | T, LM |
| keratin, type I cytoskeletal 18 (KRT18) | 254540068 | 1.372 | 0.0006 |  | 1.933 | 0.0012 | C, EVE, | CSO |
| keratin, type II cytoskeletal 8 (KRT8) | 114145561 | 1.310 | 0.0000 |  | 1.641 | 0.0000 | C, EVE, CCJ | CSO |
| annexin A5 (ANXA5) | 6753060 | 4.056 | 0.0000 |  | 1.980 | 0.0000 | C, EPR, N | CaT, ST |
| RAB1A, member RAS oncogene family (RAB1A) | 6679587 | 1.949 | 0.0009 |  | 0.489 | 0.0004 | C, GA, EPR | Aph, ST, T |
| acyl-CoA oxidase 1(ACOX1) | 66793429 | 0.412 | 0.0000 |  | 0.422 | 0.0000 | C, P, N | LM, FABO |
| acyl-CoA oxidase 2 (ACOX2) | 239787090 | 0.723 | 0.0198 |  | 0.487 | 0.0090 | C, P | LM, FABO |
| acetyl-CoA acetyltransferase 2 (ACAT2) | 148747461 | 0.766 | 0.0041 |  | 0.845 | 0.0113 | C, P, EPR, N | LM, FABO |
| peroxisomal membrane protein 2 (PXMP2) | 112421058 | 0.567 | 0.0000 |  | 0.649 | 0.0001 | C, P | RROS |
| ornithine carbamoyltransferase, mitochondrial (OTC) | 6679184 | 0.492 | 0.0000 |  | 1.094 | 0.1000 | C, M | UC, ABP |
| argininosuccinate lyase (ASL) | 19526986 | 0.828 | 0.0127 |  | 1.075 | 0.1100 | C, M, EPR, N | UC, ABP, A |
| argininosuccinate synthase (ASS1) | 6996911 | 0.863 | 0.0140 |  | 2.018 | 0.0000 | C, M, EPR, N | UC, ABP |
| arginase-1, liver (ARG1) | 7106255 | 0.509 | 0.0000 |  | 0.722 | 0.0000 | C, M, ES | UC, ACP |
| glutamate dehydrogenase 1 (GLUD1) | 6680027 | 1.277 | 0.0009 |  | 1.875 | 0.0000 | C | AAM, ABP |
| malate dehydrogenase 1, cytoplasmic (MDH1) | 254540027 | 0.717 | 0.0000 |  | 0.890 | 0.0170 | C, P | CMP, GNG, ORP |
| dimethylglycine dehydrogenase (DMGDH) | 21311901 | 0.554 | 0.0000 |  | 0.794 | 0.0000 | C, M | ChM, ORP, BCP |
| glutamic-oxaloacetic transaminase 1 (GOT1) | 160298209 | 0.613 | 0.0219 |  | 0.550 | 0.0011 | C, M | AAM, UC, TCA, SMD |
| methionine adenosyltransferase 1A (MAT1A) | 19526790 | 0.719 | 0.0000 |  | 0.896 | 0.0009 | C, N | AAM, Met, SMD |
| adenosylhomocysteinase (AHCY) | 262263372 | 0.584 | 0.0000 |  | 0.713 | 0.0000 | C, N | HBP, Met |
| betaine-homocysteine S-methyltransferase 1 (BHMT) | 7709990 | 0.256 | 0.0000 |  | 0.277 | 0.0000 | C, EPR | ChM, Met, BCP |
| glycine N-methyltransferase (GNMT) | 6754026 | 0.483 | 0.0000 |  | 0.750 | 0.0000 | C | CNCMP, Met, MMP |
| catechol O-methyltransferase (COMT) | 161484638 | 0.855 | 0.0100 |  | 0.919 | 0.1350 | C, EVE, M | CatMP, Met |
| serine hydroxymethyltransferase 1 (SHMT1) | 67846103 | 0.895 | 0.0580 |  | 0.865 | 0.0650 | C, EVE, M, N | AAM, GBPS |
| pyruvate carboxylase (PC) | 251823980 | 0.818 | 0.0100 |  | 1.055 | 0.4000 | C, M | GNG, LM |
| hydroxypyruvate isomerase (putative) (Hyi) | 342349351 | 0.140 | 0.0000 |  | 0.166 | 0.0001 | U | CM, T |
| pyridoxal kinase (PDXK) | 26006861 | 0.837 | 0.0410 |  | 0.317 | 0.0002 | C, ES, N | PPB, CP, NRAP |
| catalase (CAT) | 157951741 | 0.819 | 0.0100 |  | 0.824 | 0.0000 | C, EPR, ES | OSR, ChM |
| glucose-6-phosphate isomerase (GPI) | 254553458 | 1.858 | 0.0000 |  | 1.381 | 0.0000 | C, EVE, ES | G, GNG, CMP |
| fructose-1,6-bisphosphatase 1 (FBP1) | 9506589 | 1.110 | 0.0500 |  | 1.544 | 0.0000 | C, EVE, N | CMP, GNG, FMP |
| phosphoglycerate mutase 1 (PGAM1) | 114326546 | 1.418 | 0.0000 |  | 1.430 | 0.0000 | C, EVE | G, GNG |
| pyruvate kinase PKLR isoform 1 (PKLR) | 153792131 | 2.014 | 0.0018 |  | 1.337 | 0.0026 | C, EVE | ATPBP, CMP |
| citrate synthase (CS) | 13385942 | 1.739 | 0.0000 |  | 1.356 | 0.0004 | EVE, M, N | TCA, CMP |
| glutamine synthetase (GS) | 31982332 | 4.280 | 0.0000 |  | 1.071 | 0.2420 | C, EVE, N | AAM, AAC |
| heat shock protein family A (Hsp70) member 5 (HSPA5) | 254540168 | 1.845 | 0.0000 |  | 0.872 | 0.0100 | C, EPR, GA, M | PF, UPR |
| carbonic anhydrase 2 (CA2) | 157951596 | 1.504 | 0.0000 |  | 1.177 | 0.0164 | C, PM | ST |
| protein disulfide-isomerase A6 precursor (PDIA6) | 377833208 | 2.765 | 0.0000 |  | 1.132 | 0.0100 | C, PM | ORP, PF |
| UDP-glucose 6-dehydrogenase (UGDH) | 6678499 | 1.964 | 0.0001 |  | 1.154 | 0.0313 | C, EVE, N | ORP, UDPGMP |
| peroxiredoxin-4 (PRDX4) | 7948999 | 1.880 | 0.0005 |  | 0.230 | 0.0002 | C, ER | ORP, EMO |
| sulfide:quinone oxidoreductase, mitochondrial (SQOR) | 244790049 | 1.592 | 0.0000 |  | 3.234 | 0.0000 | C, M | HSMP |
| epoxide hydrolase 1 (EPHX1) | 6753762 | 2.095 | 0.0000 |  | 1.143 | 0.0200 | C, ES, EVE, M | XM, Nrf2OSR |
| aldehyde dehydrogenase 2 (ALDH2) | 6753036 | 1.537 | 0.0001 |  | 2.185 | 0.0000 | C, M | FAAO, PD III |
| aldehyde dehydrogenase 3 family member A2 (ALDH3A2) | 75677435 | 1.733 | 0.0000 |  | 1.094 | 0.0510 | PM, EPR | LPS/IL-1 MI RXR,ST |
| cytochrome P450 1A2 (CYP1A2) | 6753566 | 2.026 | 0.0000 |  | 0.821 | 0.0100 | C, M, EPR | XM, PXR/RXR S |
| cytochrome P450 2A5 (CYP2A5) | 75832129 | 7.820 | 0.0001 |  | 1.533 | 0.0028 | C, M, N | LPS/IL-1 MI RXR, |
| cytochrome P450 51A1 (CYP51A1) | 71061451 | 1.393 | 0.0001 |  | 1.001 | 0.5004 | C, M | LXR/RXR A, LB |
| glutathione S-transferase A2 (GSTA2) | 50263046 | 2.245 | 0.0128 |  | 1.216 | 0.0140 | C | GMP |
| glutathione S-transferase Mu 1 (GSTM1) | 6754084 | 2.365 | 0.0000 |  | 1.149 | 0.0100 | C | LPS/IL-1 MI RXR, XM |
| glutathione S-transferase Mu 2 (GSTM2) | 6680121 | 2.647 | 0.0000 |  | 0.809 | 0.0100 | C | LPS/IL-1 MI RXR, XM |
| glutathione S-transferase Mu 3 (GSTM3) | 33468899 | 9.694 | 0.0011 |  | 2.853 | 0.0010 | C | LPS/IL-1 MI RXR, XM |
| glutathione peroxidase 1 (GPX) | 84871986 | 0.653 | 0.0000 |  | 1.392 | 0.0007 | C | GMP, GRR |
| glutathione S-transferase kappa 1 | 21313138 | 0.665 | 0.0000 |  | 0.873 | 0.0100 | C, M | GMP, GPX Activity |
| glutathione S-transferase P1 (GSTP1) | 10092608 | 0.521 | 0.0000 |  | 0.599 | 0.0021 | C | XM, GMP |
| microsomal glutathione S-transferase 1 (MGST1) | 31981068 | 0.714 | 0.0000 |  | 0.946 | 0.0464 | C, ER, M | GMP, GPX Activity |
| fatty acid synthase (FAS) | 93102409 | 1.769 | 0.0000 |  | 1.064 | 0.1000 | C | LXR/RXR A, ST, LB |
| microsomal triglyceride transfer protein (MTTP) | 254540023 | 1.483 | 0.0000 |  | 0.879 | 0.0100 | ER, GA | LM, ChM |
| phosphatidylethanolamine-binding protein 1 (PEBP1) | 84794552 | 1.796 | 0.0252 |  | 1.445 | 0.0034 | C | PLCS |
| fatty acid-binding protein, liver (FABP) | 8393343 | 0.799 | 0.0000 |  | 0.763 | 0.0000 | C | LM, LT, FABO |
| alanyl-tRNA synthetase (AARS) | 34610207 | 2004 | 0.0000 |  | 1.001 | 0.2310 | C | tRNA charging |

ACS, actin cytoskeleton; C, cytoplasm; CCJ, cell-cell junction; ECD, epithelial cell differentiation; ES, extracellular space; EPR, endoplasmic reticulum; EVE, extracellular vesicular exosome; FA, focal adhesions; GA, Golgy apparatus; IM, integral to membrane; IFCS, intermediate filament cytoskeleton; IS, intracellular space; L, lysosome; M, mitochondrion; MS, microsome; N, nuclei; P, peroxisome; PM, plasma membrane; PP, pseudopodia; PR, perinuclear region; U, unknown. A, aging; AAC, ammonia assimilation cycle; AAM, amino-acid metabolism; ABP, arginine biosynthetic process; ATPBP, ATP biosynthetic process; ACO, actin cytosceleton organization; ACP, arginine catabolic process; AD, adhesion; AFM, actin filament moving; AFR, acute phase response; ANG, angiogenesis; AP, apoptosis process; APh, autophagy; BCP, betaine catabolic process; CaT, calcium transport; CC, cell cycle; CDE, clathrine-dependent endocytosis; ChM, cholesterol metabolism; CJA, cell junction assembly; CaM, calcium metabolism; CaIT, calcium ion transport; CICMA, calcium-independent cell-matrix adhesion; ChM, choline metabolism; CM, cellular metabolism; CMP, carbohydrate metabolic process; CatMP, catecholamine metabolic process; CNCMP, cellular nitrogen compound metabolic process; CP, cell proliferation; CPL, cellular protein localization; CSO, cytoskeleton organization; EMO, extracellular matrix organization; EVE, estracellular vesicle exosome; FAAO, fatty acid α-oxidation; FABO, fatty acid beta-oxidation; FMP, fructose metabolic process; G, glycolysis; GB, glycogen biosynthesis; GBPS, glycine biosynthetic process from serine; GMP, glutathione metabolic process; GNG, gluconeogenesis; GRR, glutathione redox reactions; HBP, homocysteine biosynthetic process; HSMP, hydrogen sulfide metabolic process IA, integrin activation; IFO, intermediate filament cytoskeleton organization; IR, immune response; LB, lipid biosynthesis; LM, lipid metabolism; LPS/IL-1 MI RXR, LPS/IL-1 mediated inhibition of RXR function; LT, lipid transport; LXR/RXR A, LXR/RXR activation; M, migration; MC, muscle contruction; MMP, methionine metabolic process, Met, methylation; MO, mitochonrrial organization; NRAP, negative regulation of apoptotic process; Nrf2OSR, Nrf2 oxidative stress response; ORP, oxidation-reduction process; PCP, protein catabolic process; PD III, putrescine degradation III; PF, protein folding; PLCS, phospholipase C signaling; PP, peroxisomal proliferation; PPB, pyridoxal phosphate biosynthetic process; PT, protein transport; PXR/RXR S, PXR/RXR signaling; RROS, response to reactive oxygen species; RUP, response to unfolded protein; SMD, superpathway of methionine degradation; SMP, superoxide metabolic process; SMTFA, skeletal muscle thin filament assembly; ST, signal transduction; T, transport; TCA, tricarboxylic acid cycle; TL, translation; TR, transcription regulation; UC, urea cycle; UDPGMP, UDP-glucose metabolic process; UPR, unfolded protein response; XM, xenobiotic metabolism.
