# Supplementary material for: Accumulation of 8-hydroxydeoxyguanosine, L-arginine and Glucose Metabolites by Liver Tumor Cells Are the Important Characteristic Features of Metabolic Syndrome and Non-Alcoholic Steatohepatitis-Associated Hepatocarcinogenesis
Source: Int J Mol Sci. 2020 Oct 20;21(20):7746. doi: 10.3390/ijms21207746 (PMC7594076; doi:10.3390/ijms21207746)
Supplement: Supplementary file 1 [file ijms-21-07746-s001.zip › Table S2.docx]

| Table S2. Up-stream regulator analysis in TSOD mice tumors by IPA. | | | | | |
| --- | --- | --- | --- | --- | --- |
|  |  | TSOD HCC vs TSNO control liver | | | |
| Upstream Regulator | Molecule Type | Predicted Activation State | Activa-tion z-score | p-value | Target molecules in dataset |
| D-glucose | chemical - endogenous mammalian | Activated | 2.186 | 5.94E-06 | 3-hydroxy-3-methylglutaryl-coenzyme A, acetoacetyl-coenzyme A, acetyl-coenzyme A,ADP, ATP, citric acid, creatine, cyclic AMP, GDP, glucose-6-phosphate, glutathione, glutathione disulfide, GTP, L-aspartic acid, L-glutamic acid, NADH, NADPH, pyruvic acid, sn-glycerol-3-phosphate |
| Creatine | chemical - endogenous mammalian | Activated | 2.000 | 0.000199 | AMP, ATP, creatine, creatinine, GDP, NAD+, phosphocreatine |
| © 2000-2019 QIAGEN. All rights reserved. | | | | |  |
