# Supplementary material for: Accumulation of 8-hydroxydeoxyguanosine, L-arginine and Glucose Metabolites by Liver Tumor Cells Are the Important Characteristic Features of Metabolic Syndrome and Non-Alcoholic Steatohepatitis-Associated Hepatocarcinogenesis
Source: Int J Mol Sci. 2020 Oct 20;21(20):7746. doi: 10.3390/ijms21207746 (PMC7594076; doi:10.3390/ijms21207746)
Supplement: Supplementary file 1 [file ijms-21-07746-s001.zip › Table S1.docx]

| Table S1. Organ weights and blood biochemistry analysis in TSOD and TSNO mice. | | |
| --- | --- | --- |
| Group | TSOD | TSNO |
| No. of mice ^a)^ | 16 | 5 |
| Final body weight (g) | 64.0±2.7^*^ | 40.6±3.7 |
| Organ weighs (%) |  |  |
| Liver | 4,6±0,8^*^ | 3,6±0,2 |
| Kidneys | 1,2±0,1^*^ | 1,5±0,2 |
| Spleen | 0,28±0,08 | 0,24±0,04 |
| Fasting blood glucose (mg/dl) | 269.50±25.55^***^ | 97.8±2.58 |
| Glucose in urine (mg/dl) | 3.65±0.17^*^ | 2.87±4.03 |
| Triglicerides (mg/dl) | 184±48^***^ | 68±30 |
| Total cholesterol (mg/dl) | 254±39^***^ | 134±26 |
| Free fatty acids (mEq/l) | 3.00±0.56^*^ | 2.30±0.46 |
| AST (U/l) | 226±105^***^ | 73±18 |
| ALT (U/l) | 102±45^***^ | 28±4 |
| ALP (U/l) | 911±384^***^ | 174±23 |
| Total bilirubin (mg/dl) | 0.13±0.04 | 0.10±0.00 |
| Albumin (g/dl) | 3.44±0.41^*^ | 2.98±0.19 |

*P<0.05, *P<0.01; ***P<0.0001 as compared to the respective controls; ^a)^ Final number of the survived mice
